# Dual Function Behavior of Carbon Fiber-Reinforced Polymer in Simulated Pore Solution

**DOI:** 10.3390/ma9020103

**Published:** 2016-02-06

**Authors:** Ji-Hua Zhu, Guanping Guo, Liangliang Wei, Miaochang Zhu, Xianchuan Chen

**Affiliations:** Guangdong Province Key Laboratory of Durability for Marine Civil Engineering, School of Civil Engineering, Shenzhen University, Shenzhen 518060, Guangdong, China; zhujh@szu.edu.cn (J.-H.Z.); guoguanping@email.szu.edu.cn (G.G.); weiliangliang@email.szu.edu.cn (L.W.); zhumiaochang@gmail.com (M.Z.)

**Keywords:** polymer-matrix composites (PMCs), corrosion, electrical properties, mechanical properties, anode

## Abstract

The mechanical and electrochemical performance of carbon fiber-reinforced polymer (CFRP) were investigated regarding a novel improvement in the load-carrying capacity and durability of reinforced concrete structures by adopting CFRP as both a structural strengthener and an anode of the impressed current cathodic protection (ICCP) system. The mechanical and anode performance of CFRP were investigated in an aqueous pore solution in which the electrolytes were available to the anode in a cured concrete structure. Accelerated polarization tests were designed with different test durations and various levels of applied currents in accordance with the international standard. The CFRP specimens were mechanically characterized after polarization. The measured feeding voltage and potential during the test period indicates CFRP have stable anode performance in a simulated pore solution. Two failure modes were observed through tensile testing. The tensile properties of the post-polarization CFRP specimens declined with an increased charge density. The CFRP demonstrated success as a structural strengthener and ICCP anode. We propose a mathematic model predicting the tensile strengths of CFRP with varied impressed charge densities.

## 1. Introduction

It is well known that the corrosion of steel is the major cause of damage of reinforced concrete structures exposed to de-icing salts or marine environments [1,2]. Traditional techniques for local repair are not only laborious and interferential with operational service, but are also not effective in reducing the corrosion rate [3]. The impressed current cathodic protection (ICCP) technique is currently well accepted as a suitable technique for the protection of reinforced concrete structures that have been damaged by chloride-induced corrosion [4]. The Federal Highway Administration even suggested that the only effective corrosion prevention method for a contaminated reinforced concrete member is the ICCP method [5].

Due to concrete’s high resistivity and the complex geometry of reinforcing bars, an important consideration in the ICCP system is the selection of a suitable anode material [1,6]. A number of anode systems have currently been investigated in the impressed cathodic protection, including activated titanium mesh [7], metalized zinc [8], conductive organic paints [9], and coating-overlay anodes [10]. However, these anode systems are either highly expensive or suffer from durability problems. Furthermore, they are not compatible with concrete materials. For instance, conductive paints are cheaper, but they cannot supply current densities higher than 20 mA/m^2^ for long periods of time [10]. The development of new types of anodes for the protection of reinforced concrete structures is a subject of great technological interest.

A successful ICCP system should keep the anode material electrochemically stable while reducing its corrosion rate to a low level [6,11]. The accelerated testing of anodes for use in concrete is commonly adopted, with the purpose of indicating the anode’s ability to perform satisfactorily for a specific number of years. Unfortunately, accelerated life testing cannot be conducted in concrete, because testing at high current levels causes premature failure through electrolysis of the concrete. Therefore, accelerated life testing must be conducted in an aqueous solution, as specified in NACE Standard TM0294-2007 [12].

Carbon fiber-reinforced polymers (CFRP) consist of strong and light carbon fibers embedded in a polymer matrix. The remarkable mechanical properties suggest that CFRP is an ideal material for structurally strengthening reinforced concrete structures [13]. Structural strengthening is generally achieved by warping or adhering CFRP to the surface of reinforced structures using contact materials, thereby improving the load-carrying capacity of degraded structures [14,15]. CFRP is conductive with a polarization potential close to that of noble metals, which may induce galvanic corrosion in steel when CFRP is used to structurally strengthen steel structures [16]. Therefore, the feasibility of CFRP as an anode material in ICCP systems is an interesting problem. Moreover, the excellent conductivity, high mechanical strength, and lower cost of CFRP would present a novel and affordable method to improve the durability of reinforced concrete structures with CFRP simultaneously acting as the ICCP anode and structural strengthening material.

Studies have been performed on the dual-functional behavior of CFRP on reinforced concrete. Lee-Orantes *et al.* [17] experimentally investigated the use of CFRP anodes in the ICCP systems of reinforced concrete prisms. Nguyen *et al.* [18] studied the electrochemical performance of CFRP fabric and rods in both a calcium solution and in concrete. In addition, CFRP was employed in pre-corroded reinforced concrete beams for both structural strengthening and ICCP; the results showed that the ultimate strength of CFRP for dual functions was slightly lower than it was for a control specimen, in which CFRP was used for structural strengthening only [6].

The above works investigated the corrosion and mechanical behavior of steel and concrete in ICCP systems with CFRP anodes. However, extensive study on the electrochemical performance of CFRP itself is also important. According to the NACE Standard TM0294-2007 [12], accelerated anode testing should be conducted in three specified aqueous solutions of NaCl, NaOH, and a pore solution to characterize the resistance of the anode material to tolerate chloride evolution, oxygen evolution, as well as the actual concentrations of the pore water components and any possible synergistic effects imposed by these components, respectively. In the previous study [19,20], Zhu *et al.* investigated CFRP’s mechanical and electrochemical performance during accelerated polarization in NaCl and NaOH solution environment. It was shown that CFRP can be successfully used as the anode in an ICCP system without significant degradation of mechanical properties in NaCl and NaOH solution environment [21]. Following these studies, this work presents a systematic investigation, including the electrochemical behavior, mechanical strength, and failure modes of CFRP during anodic polarization in a simulated pore solution environment. CFRP’s electrochemical performance during anodic polarization with the pore solution was investigated using a simulated ICCP system. The mechanical strengths and failure modes of the CFRP specimens were obtained after the polarization tests and the relationship between the tensile strengths and impressed charge densities was elucidated.

## 2. Experimental Details

### 2.1. Materials

**Figure 1:**
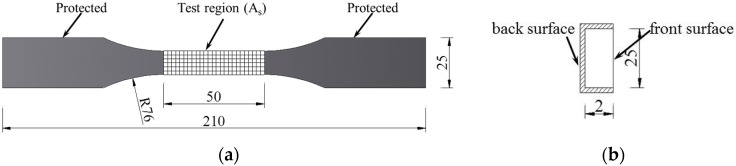
Geometric dimensions of carbon fiber-reinforced polymer (CFRP) specimens. (**a**) Front view; (**b**) Sectional view (unit: mm).

The CFRP strips (CA.BEN Composite Co., Ltd., Hong Kong) were made from multi-layer carbon fibers (Toray T700, supplied by CA.BEN Composite Co., Ltd., Hong Kong) with a volume fraction of 60% embedded in LAM-125/LAM-226 epoxy (Pro-Set Inc., Bay City, MI, USA). The chemical composition of the epoxy used in the CFRP is shown in Table 1. CFRP specimens were machined into dumbbell shapes for mechanical testing according to the ASTM Standard D638-10 [22], as shown in Figure 1a. Specimens were protected by Kafuter K-5704RTV sealant (Guangdong Hengda New Materials Technology Co., Ltd., Guangdong, China), except for the front surface of the test region in the center, as shown in Figure 1b, with a nominal anodic surface area of 650 mm^2^ for the anodic polarization tests. The geometric dimensions of the CFRP specimens are detailed in Figure 1.

**Table 1:** Chemical composition of epoxy in CFRP.

Ingredients	Concentration (%)
Bisphenol-A type epoxy resin	37–38
Novolac epoxy resin	19–20
Dicyandiamide	5–6
Methyl ethyl ketone (MEK)	36–37

### 2.2. Testing Methods

#### 2.2.1. Accelerated Polarization Test

**Figure 2:**
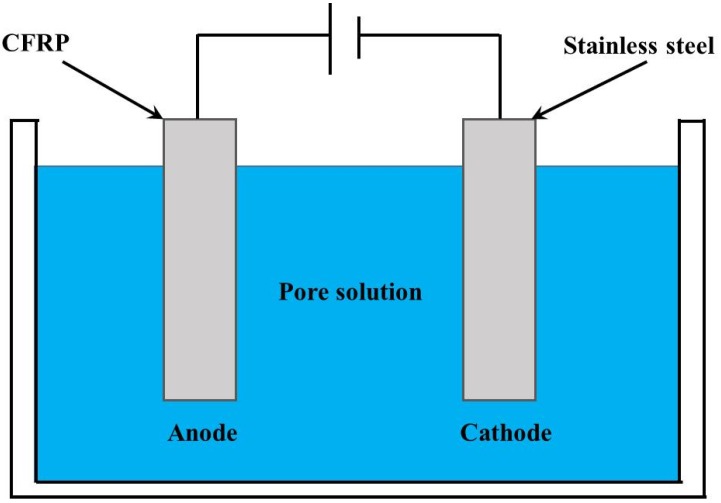
Schematic view of the simulated impressed current cathodic protection (ICCP) system.

Accelerated polarization tests were conducted to investigate CFRP’s galvanostatic anodic polarization behavior in a simulated ICCP system (as shown in Figure 2)—including an impressed current anode of CFRP, a cathode of a stainless steel strip, an aqueous pore electrolyte solution, a power source, and a saturated calomel electrode (SCE). The composition of the simulated pore solution is shown in Table 2. The anodic polarization of CFRP was achieved by connecting the CFRP specimen to the positive terminal of the power source. The exposed area of the stainless steel cathode was equal to the CFRP test area (*A_s_*) to obtain a uniform electric field distribution between the anode and cathode.

**Table 2:** The composition of the simulated pore solution.

Ingredients	Concentration (%)
Ca(OH)_2_	0.2
KCl	3.2
NaOH	2.45
KOH	1
Distilled water	93.15

A constant current supply was used to achieve galvanostatic anodic polarization. Currents of 0, 0.5, 1, 2, and 4 mA were applied, with corresponding nominal current densities of 0, 0.77, 1.54, 3.08, and 6.15 A/m^2^, respectively. Each current was tested for two distinct anodic polarization durations of 25 and 50 days. Hence, a total of ten conditions were investigated.

The specimens were labeled according to the applied currents and the anodic polarization durations for ease of identification, as shown in Table 3. For example, the label I0.5D25# indicates that the specimen was exposed to the nominal applied current, *I*, of 0.5 A for a test duration, *D,* of 25 days. Two duplicate specimens were tested for each condition; the # symbol indicates the second specimen. Table 3 also shows the critical parameters used for the post-polarization tensile tests. The I0.5D50# specimen is not shown in Table 3 because the extensometer demonstrated problems during the test process.

**Table 3:** Parameters and test results for specimens used for tensile tests after polarization.

Specimen	*A_s_* (mm^2^)	*A_c_* (mm^2^)	*i* (A/m^2^)	*q* (10^7^ C/m^2^)	*f*_u_ (MPa)	Failure Modes	*K*_Exp_	*K*_Cal_/*K*_Exp_
I0D25	670.40	25.56	0	0	774.79	L	–	–
I0D25#	669.63	25.66	0	0	649.71	L	–	–
I0.5D25	642.64	25.19	0.889	0.192	675.58	L	0.99	0.92
I0.5D25#	616.17	25.30	0.926	0.200	699.42	L	1.02	0.89
I1D25	578.82	26.18	1.888	0.408	559.89	L	0.82	1.00
I1D25#	657.50	25.97	1.637	0.354	549.92	L	0.80	1.05
I2D25	646.07	25.71	3.085	0.666	513.21	D	0.75	0.96
I2D25#	656.75	26.27	3.024	0.653	477.16	D	0.70	1.04
I4D25	656.25	25.27	6.066	1.310	474.00	D	0.69	0.76
I4D25#	630.48	26.07	6.355	1.373	466.77	D	0.68	0.75
I0D50	671.16	25.23	0	0	682.09	L	–	–
I0D50#	668.61	25.79	0	0	627.16	L	–	–
I0.5D50	644.60	25.45	0.887	0.383	653.59	L	0.96	0.87
I1D50	657.25	25.70	1.611	0.696	318.15	D	0.47	1.53
I1D50#	643.62	24.83	1.666	0.720	340.22	D	0.50	1.41
I2D50	656.75	25.09	2.981	1.288	329.08	D	0.48	1.11
I2D50#	630.72	25.69	3.110	1.344	303.17	D	0.44	1.17
I4D50	632.64	26.23	6.354	2.745	193.49	D	0.28	0.93
I4D50#	618.76	26.00	6.424	2.775	181.88	D	0.27	0.97
Mean	–	–	–	–	–	–	–	1.02
COV	–	–	–	–	–	–	–	0.211

Notes: # = duplicate specimen. *A_s_* = measured anodic surface area, *A_c_* = measured cross-sectional area, *i* = actual current densities, *q* = charge density, *f*_u_ = ultimate tensile strength, L= lateral failure type, D= edge delamination failure type, *K*_Exp_ = experimental deterioration factor, *K*_Cal_ = calculated deterioration factor.

The anodic performance of CFRP was evaluated by recording the feeding voltage between the CFRP and stainless steel. The potential of CFRP *versus* the SCE was also measured during the galvanostatic anodic polarization. The feeding voltage between CFRP and stainless steel, together with the potential of CFRP, were monitored every 10 min throughout the test period with a multi-channel data logger.

#### 2.2.2. Tensile Test

Uniaxial tensile tests were conducted on the CFRP specimens after the galvanostatic anodic polarization. Tensile tests were performed on a universal test machine (E45, MTS, Eden Prairie, MN, USA) at a constant loading rate of 0.2 mm/min. The applied load and displacement between the machine grips for each test were recorded using a data acquisition system. The strains of the specimens were not measured, as it was not possible to attach strain gauges to the heavily corroded specimen surfaces.

## 3. Results and Discussion

### 3.1. Anode Performance

**Figure 3:**
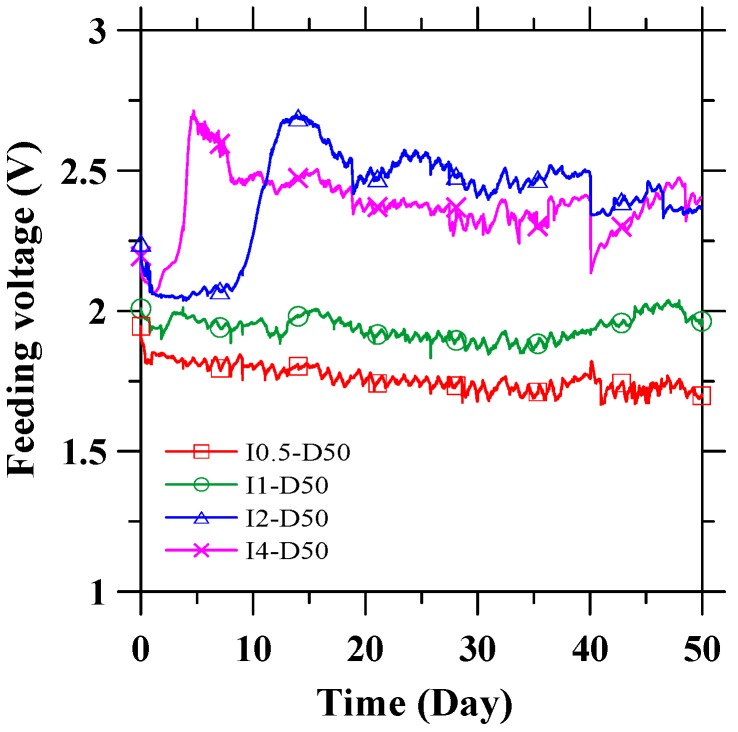
The feeding voltage between CFRP and stainless steel during galvanostatic anodic polarization process.

The feeding voltages between the CFRP anode and the steel cathode were used to demonstrate the variation of resistance in the electrical circuit, as a constant current was applied throughout the test via the pore solution. Figure 3 depicts the recorded feeding voltages between the CFRP and stainless steel during galvanostatic anodic polarization for 50 days. At all given currents, the feeding voltages gradually stabilized at values between 1.7 and 2.4 V with increased galvanostatic anodic polarization time. This trend indicates the stable and serviceable performance of CFRP as the impressed current anode in an ICCP system.

**Figure 4:**
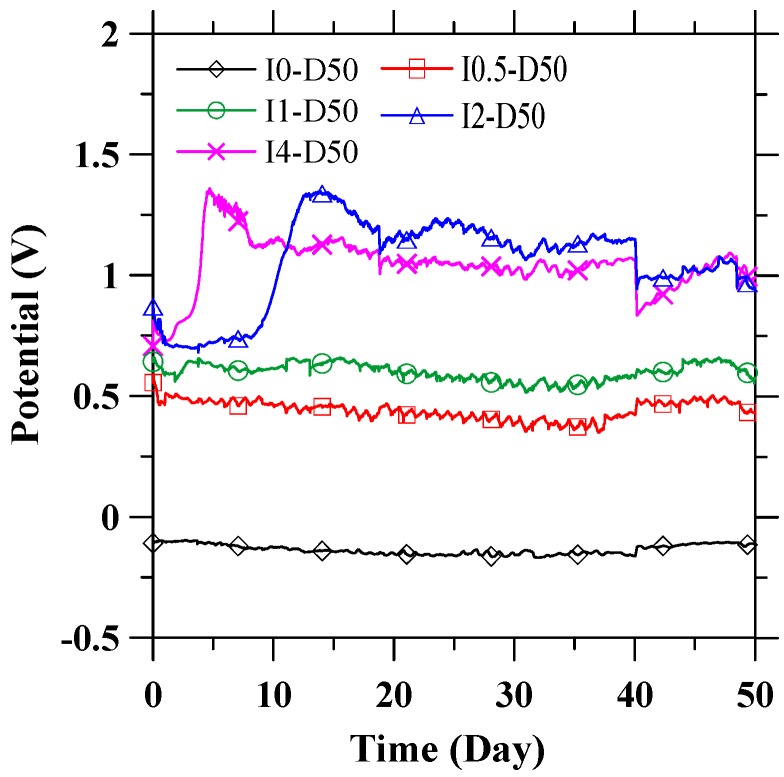
The potential (*vs.* SCE) of CFRP during galvanostatic anodic polarization process.

The potentials of the CFRP anode reflect the anodic performance during the galvanostatic anodic polarization process. The potential of CFRP with respect to the SCE during the tests are presented in Figure 4. Specimen I0-D50, which was immersed in the electrolyte solution for 50 days without an applied current, is shown in Figure 4 for reference. The potential of CFRP shows a trend similar to that of the feeding voltage between CFRP and stainless steel. The potential of anodically polarized CFRP is within the range of 0.4–1.2 V (*vs.* SCE), while the potential of specimen I0-D50 (without external current) is approximately −0.135 V (*vs.* SCE). The stabilizing potential curve demonstrates the stable anodic performance of CFRP during anodic polarization in the pore solution. Figure 3 and Figure 4 show a drop in both the feeding voltage and potential at around the 40th day. This drop is caused by an accidental interruption of supplied power. However, the feeding voltage and potential were recovered once the power supply was restored.

### 3.2. Tensile Strength and Failure Modes

The mechanical strengths of CFRP specimens obtained from tensile tests after polarization are shown in Table 3. The tensile strength of each specimen was calculated as the ultimate applied load over the pre-polarization cross-sectional area, because the post-polarization corroded specimen surfaces prevented accurate measurements. The tensile strengths of the CFRP specimens decrease as the current density increases. The tensile strengths also decrease with longer polarization periods, even at the same impressed current density. The specimens without an applied current (I0D25, I0D25#, I0D50, and I0D50#) had an average tensile strength of 683.44 MPa, while the average tensile strength was reduced to 187.69 MPa for specimens I4D50 and I4D50# at a current density of 6.389 A/m^2^ applied for 50 days.

**Figure 5:**
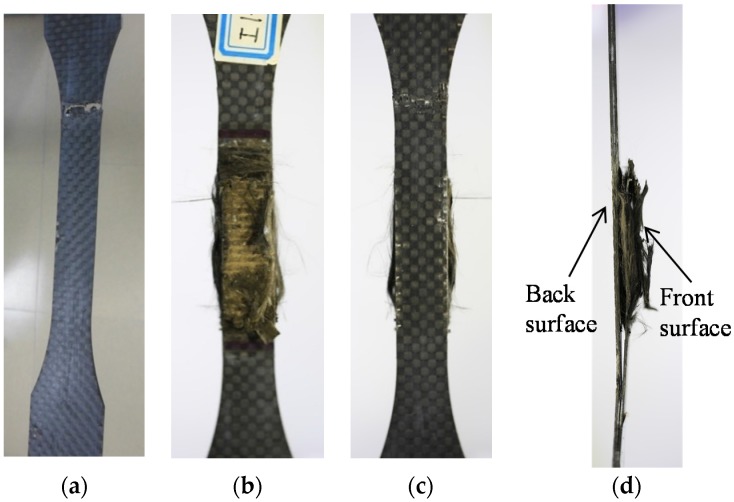
Failure modes of CFRP specimens obtained from tensile tests. (**a**) L (Lateral) failure mode; (**b**) D (edge Delamination) failure mode, front surface; (**c**) D (edge Delamination) failure mode, back surface; and (**d**) D (edge Delamination) failure mode, side view.

Two typical failure modes of the CFRP specimens during uniaxial tensile tests were observed, as shown in Table 3 and Figure 5. The first caused a lateral failure across the gauge lengths of the specimens, as shown in Figure 5a. The second demonstrated a vertical failure along the specimen lengths with edge delamination, as shown in Figure 5b–d. According to ASTM Standard D3039/D3039M [23], the two failure modes are defined as lateral (L) and edge delamination (D) modes.

Specimens I0D25, I0.5D25, I1D25, I0D50, and I0.5D50, with lower applied current densities and shorter polarization periods, experienced L mode, while specimens with higher applied current densities and longer polarization periods experienced D mode. The L failure mode is similar to the typical mechanical failure of CFRP, according to numerous reports [24]. It is evident that the specimens were not seriously degraded by polarization. However, with increased currents and duration, more serious degradation of CFRP occurred at the test region. Figure 5b,c show the front and back surfaces of a polarized CFRP sample with D failure mode. Notably, the front surface of the specimen is in contact with the solution for polarization, while the back surface is protected with sealant. Figure 5d shows the side view of the same sample. It is apparent that the left side, which corresponds to the specimen’s back surface, is in good condition, and the epoxy matrix retained. However, the specimen’s right side (front surface) is so deeply corroded that the epoxy matrix has been almost destroyed during polarization. Therefore, D mode is a delamination failure associated with the degradation of the epoxy polymer at the anode region of the specimens.

The degradation mechanism of the CFRP mechanical properties may be associated with the depolymerization of epoxy polymer generated at the anode region. Zhu *et al*. [19] investigated The electrochemical performance and mechanical properties of CFRP during anodic polarization using NaCl solution. The results show that the epoxy in CFRP was corroded during anodic polarization, which caused the observed degradation in mechanical properties. The degradation of the epoxy in NaCl solution may be attributed to the breakage of C-N bonds, which caused the depolymerization of the epoxy. It is believed that CFRP with a higher charge density is associated with greater depolymerization of the epoxy polymer. The epoxy’s degradation in the pore solution may be attributed to the breakage of C-N bonds, which caused the depolymerization of the epoxy.

### 3.3. Relationship between Tensile Strength and Charge Density

**Figure 6:**
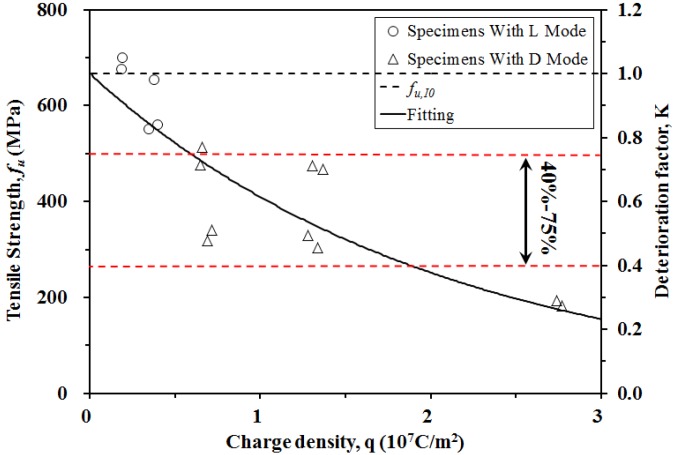
The relationship between tensile strength, K and charge density for CFRP specimens with pore solution subjected to anodic polarization.

Figure 6 shows the tensile strength of CFRP specimens plotted against the impressed charge density (*q*), where *q* is defined as the total charge per unit area of the test region over a specific time (*q* = *i* × *t*, where *t* = 25 days and 50 days). The tensile strength of CFRP clearly decreases with increasing charge density. A model has been proposed to describe the relationship between tensile strength and charge density, as shown in Equation (1):

*f_u_ = Kf_u,I0_ =* g(*q*) *f_u,I0_* (1)

where *K* is the deterioration factor of the CFRP tensile strength through anodic polarization, and is a function of *q* (unit: 10^7^ C/m^2^); *f_u_* is the tensile strength of CFRP; *f_u,I0_* is the average tensile strength of specimens I0D25 and I0D50 (equal to 682.83 MPa in this study); and g(*q*) is expressed as Equation (2):

*K* = g(*q*) = e^−0.487*q*^ (2)

Therefore, the tensile strengths of the CFRP specimens can be derived at any given charge density using Equations (1) and (2). Table 3 compares the experimental (*K*_Exp_) and calculated (*K*_Cal_) deterioration factors for CFRP specimens with varied *q*. The mean value of the *K*_Cal_/*K*_Exp_ ratio is 1.02 with a coefficient of variation (COV) of 0.211, demonstrating agreement between the calculated and experimentally measured tensile strengths.

## 4. Discussion of Service Life

This study investigates the mechanical and electrochemical performance of CFRP for the purpose of developing a novel method to improve the load-carrying capacity and durability of reinforced concrete structures. In these structures, CFRP is employed simultaneously as the structural strengthening material and the impressed anode of the ICCP system. Therefore, it is necessary to investigate the service life of a dual-function CFRP.

The service life of an impressed current anode, as characterized by the capacity to transfer charges through the anode/electrolyte interface, can be evaluated according to the NACE specification [12]. The capacity to transfer charges is defined as the total charge quantity (*Q_anode_*) passed by the anode during the application of the ICCP system, as calculated by Equation (3):

(3) $$Q_{anode}\;=\;i_{a}\;\times \;t_{g}\;\times \;A_{a}$$

where *i_a_* is the applied anodic current density, *t_g_* is the duration of the impressed current, and *A_a_* is the anodic surface area.

Based on the tensile test results, the CFRP tensile strength clearly decreases as the charge density increases, as shown in Figure 6. However, the strength utilization percentage of CFRP in concrete structures is generally between 40% and 75%, based on previous reports [25,26,27,28]. Therefore, to successfully adopt CFRP in a dual-function system within concrete, the polarized CFRP must retain sufficient strength to satisfy requirements for structural strengthening. As shown in Figure 6, the tensile strengths of the polarized specimens with a charge density of less than 1.329 × 10^7^ C/m^2^ generally satisfy the lower limit tensile strength of 40%. Other specimens (2.760 × 10^7^ C/m^2^) have tensile strengths below the 40% criterion. Therefore, the tensile strengths of CFRP specimens with charge densities of 1.329 × 10^7^ C/m^2^ satisfy the requirement for structural strengthening. This requirement could be adapted to assess the service life of a dual-functioning CFRP acting as both an impressed anode and a structural strengthening material. The capacity of the CFRP plate to transfer charge (*Q_CFRP_*) can be calculated according to Equation (4):

(4) $$Q_{CFRP}\;=\;Q_{anode}\;=\;i_{a}\;\times \;t_{g}\;\times \;A_{a}\;=\;1.329\;\times \;10^{7}\;A_{a}\;\left(C\right)$$

The service life of a practical ICCP system depends not only on *Q_anode_*, but also on the steel reinforcement configuration, among other factors, such as the anode/concrete interfacial properties and concrete quality. This study focuses on the behavior of CFRP. Therefore, by assuming that *Q_anode_* is the governing factor for service life, the service life of an ICCP system can be evaluated based on the equilibrium of the charge quantity between the cathode *(Q_cathode_*) and anode (*Q_anode_*), as shown in Equation (5).

(5) $$Q_{cathode}\;=\;Q_{anode}$$

**Figure 7:**
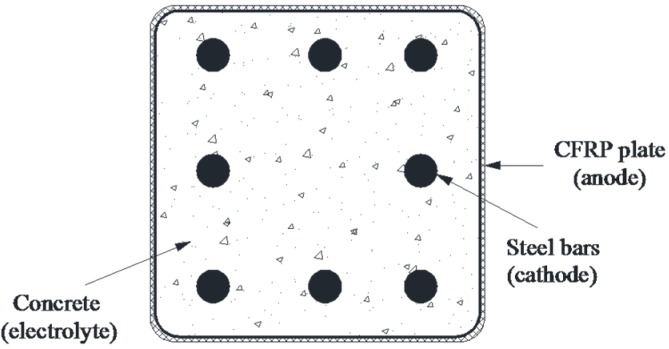
An eight-steel rebar-reinforced concrete element wrapped with CFRP plate as an anode.

The service life was investigated using a typical concrete cross-section, as shown in Figure 7. A concrete element with a cross-section of 400 × 400 mm was reinforced using eight identical lengths of steel rebar. The ICCP system was applied by wrapping a CFRP sheet around the concrete element, where the CFRP sheet and the steel rebar served as anode and cathode, respectively. Each length of steel rebar was assumed to receive identical protection current densities (*i_p_*) throughout protection, and Equations (3)–(5) were adopted. The unit length of the concrete element was considered. Therefore, the charge quantity of steel (*Q_steel_*) could be calculated using Equation (6):

(6) $$Q_{cathode}\;=\;Q_{steel}\;=\;n\;\times \;A_{steel}\;\times \;i_{p}\;\times \;t_{life}\;=\;i_{p}\;t_{life}\;\sqrt{4\pi nA_{c}\rho }$$

where *n* is the number of steel rebar lengths; *A_steel_* is the steel surface area per unit length in contact with the concrete; *i_p_* is the applied protection current density of the steel cathode; *A_c_* is the cross-sectional area of the concrete element; *ρ* is the reinforcement ratio of the concrete element, calculated by dividing the cross-sectional area of concrete by the total cross-sectional area of the steel rebar in the concrete; and *t_life_* is the service life of the ICCP system governed by *Q_CFRP_*.

Hence, *t_life_* can be calculated by inserting Equation (6) into Equation (5). The protection current density of 2–20 mA/m^2^ is recommended for an ICCP system in corrosion-deteriorated reinforced concrete structures. Protection current densities with an upper limit of 20 mA/m^2^ for ICCP, and reinforcement ratios from 0.6% to 5%, as commonly used for concrete structure design, were used in the calculation. The *t_life_* decreases as *ρ and i_p_* increase.

Table 4 shows the minimum service life of the ICCP system. When the protection current density is 20 mA/m^2^ and the reinforcement ratio is 5%, the minimum service life of 24.6 years is achieved in the pore electrolyte solution environment. The service life is roughly equal, compared to the service life in the NaCl and NaOH solutions. In these three solutions, the residual tensile strengths of CFRP minimum were only 44% when in NaOH solution.

According to the NACE Standard TM0294-2007 [12], accelerated testing shall be conducted in duplicate in the three aqueous solutions (NaCl, NaOH, and pore solution). As shown in Table 4, previous research demonstrates that CFRP plate can be successfully used as the anode material in the ICCP system over an acceptable service period, without significant degradation of the mechanical properties for structural strengthening. Notably, the predicted service life is conservative, since the polarization was conducted in a simulated ICCP system in which the corrosion environment was much more severe than it is in practical concrete structures.

**Table 4:** Comparison of the service life of the ICCP system in the different electrolyte solutions.

Electrolyte Solution Type	*q* (10^7^ C/m^2^)	*i_p_*(mA/m^2^)	*ρ*	*Average K*_Exp_	*T_min._*_life_ (Years)
NaCl solution [19]	1.331	20	5%	0.67	23.9
NaOH solution [20]	1.372	20	5%	0.44	24.6
Pore solution	1.329	20	5%	0.57	23.8

## 5. Conclusions

A comprehensive experimental program was performed to study the electrochemical and mechanical behaviors of CFRP in an impressed concrete evolution environment. Accelerated polarization tests were performed in simulated ICCP systems with simulated pore solutions. Based on this study, the following conclusions can be drawn:

1 The recorded feeding voltage indicated that an ICCP system with a CFRP anode could operate stably in a pore solution. Meanwhile, the anode performance of CFRP was maintained for 50 days of polarization with applied current densities reaching 6.15 A/m^2^, as indicated by the stabilized potential curve of CFRP.
2 The mechanical properties of the CFRP specimens were obtained from uniaxial tensile tests after anodic polarization. The tensile strengths of CFRP decreased with increased charge densities. The failure mode of CFRP after polarization transferred from lateral to edge delamination with increased impressed current densities and test durations. The impressed charge density significantly affected the mechanical properties of CFRP.
3 A model was developed based on the experimental results in order to calculate the CFRP tensile strengths at given impressed charge densities. Agreement was obtained between the calculated tensile strengths and the experimental data.
4 The service life of dual-function CFRP was discussed. In three electrolyte solutions investigated, CFRP plates were demonstrated to be capable of serving as both structural strengthening and impressed anode materials in ICCP systems for reinforced concrete structures. The minimum predicted service life was 23.9 years, 24.6 years, and 23.8 years, respectively, corresponding to the NaCl solution, the NaOH solution, and the pore solution, even with the maximum acceptable protection current density and reinforcement ratios. Notably, this prediction was conservative, because the polarization data obtained was from a simulated ICCP system with an environment much worse than that in practical concrete structures.

## Supplementary Files

Supplementary File 1

## Notation

*A_s_*: Measured anodic surface area of test region of CFRP specimens;
*A_c_*: Measured cross-sectional area of CFRP specimens;
*f_u_*: Ultimate tensile strength of CFRP specimens;
*f_u,I0_*: Average ultimate tensile strength of CFRP specimens I0D25, I0D25#, I0D50, and I0D50#;
*i*: Current density;
*q*: Charge density—the total charge per unit area passed by the CFRP surface;
*K*: Deterioration factor;
*K_Exp_*: Experimental deterioration factor;
*K_Cal_*: Calculated deterioration factor;
V: Feeding voltage or potential;
*i_p_*: Protection current density;
*n*: Number of steel rebars in the concrete element;
*Q_anode_*: Anode’s capacity to transfer charges;
*Q_cathode_*: Total charge quantity past the cathode during cathodic protection;
*Q_CFRP_*: CFRP plate’s capacity to transfer charge;
*Q_steel_*: Total charge quantity past the steel in the concrete element during cathodic protection;
*t_g_*: Duration of impressed current;
*t_life_*: Predicted service life of the ICCP system by *Q_CFRP_*;
*ρ*: Reinforcement ratio.
